# Illumina-Based Analysis Yields New Insights Into the Fungal Contamination Associated With the Processed Products of Crataegi Fructus

**DOI:** 10.3389/fnut.2022.883698

**Published:** 2022-05-12

**Authors:** Jingsheng Yu, Mengyue Guo, Wenjun Jiang, Yujie Dao, Xiaohui Pang

**Affiliations:** Institute of Medicinal Plant Development, Chinese Academy of Medical Sciences and Peking Union Medical College, Beijing, China

**Keywords:** Crataegi Fructus, high-throughput sequencing, ITS2, fungal contamination, collection areas, processing methods

## Abstract

Crataegi Fructus, a medicinal and edible herb in China, has been considered a popular dietary supplement globally. It is used for the treatment of dyspepsia and chronic heart failure according to the Chinese Pharmacopoeia (2020). However, fungal contamination in Crataegi Fructus affects its quality and safety, thus preventing its global promotion. In this study, we comprehensively studied the fungal community in processed products of Crataegi Fructus by high-throughput sequencing. A total of 21 Crataegi Fructus samples were collected from five provinces in China, and the samples were divided into five groups based on collection areas, as well as into three groups based on processing methods. We then targeted the internal transcribed spacer 2 sequence through the Illumina Miseq PE300 platform to investigate fungal composition and diversity. Results showed that all 21 samples were detected with fungal contamination, and Ascomycota was dominant at the phylum level. In the groups based on collection areas, Dothideomycetes, Pleosporaceae, and *Alternaria* were dominant at the class, family, and genus levels, respectively. In the groups based on processing methods, Dothideomycetes, Aspergillaceae, and *Alternaria* were the most abundant at the class, family, and genus levels, respectively. Differences in fungal communities between various groups were also observed. Furthermore, a total of 115 species were identified, among which seven were potential toxigenic, namely, *Trichothecium roseum*, *Alternaria tenuissima*, *Aspergillus carbonarius*, *Penicillium brevicompactum*, *Aspergillus fumigatus*, *Rhizopus microspores*, and *Pichia fermentans*. In conclusion, this study reveals great fungal richness and diversity of Crataegi Fructus, providing references for the prevention and control of fungal contamination of Crataegi Fructus in practical production.

## Introduction

As a popular dietary supplement, hawthorn has been consumed worldwide. Hawthorn is a universal name, which represents all species in the *Crataegus* genus (1). This supplement is distributed widely in Asia, Europe, and North America. In Europe, it is made into canned fruits, jams, and jellies. Furthermore, based on the European Pharmacopeia (2017), hawthorn herbal materials are derived from *Crataegus monogyna* Jacq. (Lindm.) and *Crataegus laevigata* (Poir.) DC (syn. *C. oxyacantha* L.) (2). Hawthorn is also a medicinal and edible herb in China, and its products have been made into beverages and snacks. This herb was first recorded as *Shan Zha* (in Chinese) in *Ben Cao Jing Ji Zhu* in 536 AD (3). According to the recommendation of the Chinese Pharmacopoeia (2020), Crataegi Fructus (CF) can be mainly formulated using dried, roasted, and charred products, which are derived from Cra*taegus pinnatifida* Bge. var. *major* N.E. and C*rataegus pinnatifida* Bge (4). Modern pharmacological studies have demonstrated that its extract is useful for the treatment of chronic heart failure (5). It also shows the treatment effect on high-calorie-diet-induced dyspepsia. Interestingly, compared with the effect of dried products, roasted and charred products have been demonstrated to possess a stronger curative effect (6). However, mycotoxin contamination in CF has been reported in recent years. Li et al. investigated the patulin contamination in hawthorn products, and results showed that six of 43 samples were detected to be positive, with contamination levels ranging from 19.8 to 206.88 microg/L. Meanwhile, the level of patulin in four samples exceeded the Chinese legal limit (50 microg/L) (7). Similarly, Zhou et al. also observed patulin contamination in one of 13 hawthorn samples by HPLC (8). Therefore, the quality and safety of CF have received extensive attention worldwide.

Fungal contamination in herbal materials is derived from the whole production chain, including cultivation, harvest, transport, processing, and storage. Among these procedures, processing is a crucial factor that affects the quality and safety of herbs. In order to enhance the curative effect or decrease toxicity, many herbs are processed before clinical application. Shen et al. compared the effect of stir-frying with sand and stir-frying on carbonized ginger. The result showed that the sand-fried ginger samples exhibited greater adsorption capacity than the stir-fried samples (9). Liu et al. indicated that processing methods, such as heating and water-washing, reduced the toxicity of Aconitum roots (10). In addition, processing procedures remarkably affect the fungal communities in herbs. He et al. studied the variation in fungal communities during the processing of *Polygala tenuifolia* roots. The result showed that the processing methods decreased the level of most fungal genera excluding *Penicillium* (11). Odongo et al. determined the influence of processing on the growth of fungi, indicating that cooking and fermentation methods inhibited fungal growth (12). A study performed by Guo et al. investigated the fungal community in raw and roasted Cassiae Semen samples. In comparison with raw samples, roasted samples had higher numbers of *Penicillium* and *Periconia* (13). Therefore, it is essential to assess the effect of processing methods on fungal contamination in herbs.

The development of high-throughput sequencing (HTS) provides new insights for revealing the role of microorganisms in human daily life. This method has been applied in different areas to analyze the diversity, composition, and function of microorganisms (14–16). The application of HTS for the investigation of fungal contamination in herbs exhibits some irreplaceable advantages. For example, overall fungal diversity and composition can be analyzed efficiently. In addition, some strains that cannot grow in a synthetic medium can be identified through HTS. It can also be applied to monitor the dynamic change of fungal communities in practical production (17–19). Thus, the analysis of fungal communities by HTS has become increasingly prevalent.

In this study, we firstly studied the fungal community in processed CF products using the HTS method and analyzed the differences in the fungal community between groups based on processing methods, as well as between groups based on collection areas. This study provides an efficient method for the analysis of fungal contamination in CF, thereby providing the scientific basis for its safe utilization.

## Materials and Methods

### Sampling and Treatments

**FIGURE 1:**
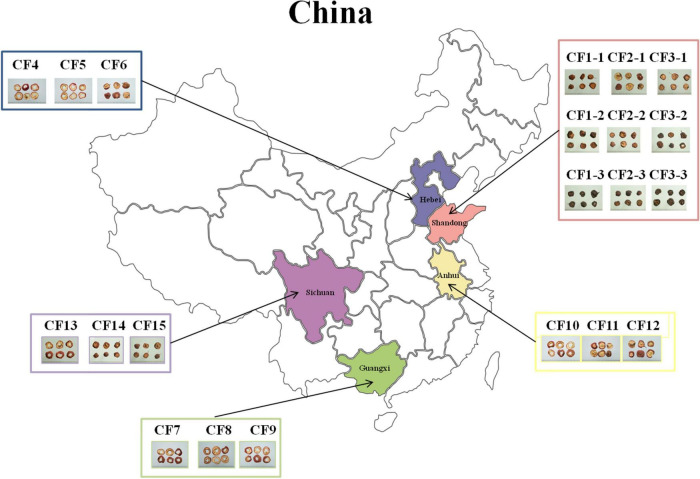
Overview of sampling in this study. A total of 21 Crataegi Fructus (CF) samples were collected from five provinces in China: Shandong (*n* = 9), Hebei (*n* = 3), Anhui (*n* = 3), Guangxi (*n* = 3), and Sichuan (*n* = 3) provinces.

In this study, we collected 21 CF samples from herbal markets in Shandong, Hebei, Anhui, Guangxi, and Sichuan provinces in China (Figure 1). Among the 21 samples, 15 dried samples were divided into five groups, namely, the SD, HB, GX, AH, and SC groups based on collection areas. Meanwhile, nine samples from Shandong province were divided into three groups, namely, the SDD (dried), SDR (roasted), and SDC (charred) groups based on processing methods.

The CF samples were processed as described by Chinese Pharmacopoeia (2020). Samples in the SDD group were dried with hot air or under the sun. The CF samples in the SDR group were processed by low-temperature heating (150°C) until they became brown. The CF samples in the SDC group were processed by medium-temperature heating (180°C) until they became brown and black. Each sample was collected with 500 g, and placed into 21 sterile paper bags, respectively. All the samples were assigned with voucher numbers and deposited in the Institute of Medicinal Plant Development, Chinese Academy of Medical Sciences (Table 1).

**TABLE 1:** Information for the Crataegi Fructus samples in this study.

Voucher No.	Sampling location	Collection time	Collection temperature	Group 1	Group 2	Processing method	Genbank accession No.
CF1-1	Shandong	2020.9	35°C	SD	SDD	Dried	SAMN18864574
CF2-1	Shandong	2020.9	35°C	SD	SDD	Dried	SAMN18864575
CF3-1	Shandong	2020.9	35°C	SD	SDD	Dried	SAMN18864576
CF1-2	Shandong	2020.9	35°C	/	SDR	Roasted	SAMN18864577
CF2-2	Shandong	2020.9	35°C	/	SDR	Roasted	SAMN18864578
CF3-2	Shandong	2020.9	35°C	/	SDR	Roasted	SAMN18864579
CF1-3	Shandong	2020.9	35°C	/	SDC	Charred	SAMN18864580
CF2-3	Shandong	2020.9	35°C	/	SDC	Charred	SAMN18864581
CF3-3	Shandong	2020.9	35°C	/	SDC	Charred	SAMN18864582
CF4	Hebei	2020.9	30°C	HB	/	Dried	SAMN18864583
CF5	Hebei	2020.9	30°C	HB	/	Dried	SAMN18864584
CF6	Hebei	2020.9	30°C	HB	/	Dried	SAMN18864585
CF7	Guangxi	2020.9	33°C	GX	/	Dried	SAMN18864586
CF8	Guangxi	2020.9	33°C	GX	/	Dried	SAMN18864587
CF9	Guangxi	2020.9	33°C	GX	/	Dried	SAMN18864588
CF10	Anhui	2020.9	33°C	AH	/	Dried	SAMN18864589
CF11	Anhui	2020.9	33°C	AH	/	Dried	SAMN18864590
CF12	Anhui	2020.9	33°C	AH	/	Dried	SAMN18864591
CF13	Sichuan	2020.9	32°C	SC	/	Dried	SAMN18864592
CF14	Sichuan	2020.9	32°C	SC	/	Dried	SAMN18864593
CF15	Sichuan	2020.9	32°C	SC	/	Dried	SAMN18864594

### Total DNA Extraction and Polymerase Chain Reaction Amplification

Total community DNA was extracted from 2.7 g CF samples using an EZNA^®^ soil DNA kit (Omega Bio-tek., Inc., Norcross, GA, United States) based on the manufacturer’s protocol. All the CF samples were transferred into a 50-ml sterilized centrifuge tube with 30 ml 1 × phosphate-buffered saline (PBS) (Beijing Solarbio Science and Technology Co., Ltd), and shaken with a cortex mixer for 5 min. In order to remove impurities, the mixture was filtered through double layers of sterile gauze. All filtrates were centrifugated at 12,000 rpm for 28 min to collect fungal strains for total DNA extraction. The DNA was stored at −20°C.

The ITS2 sequence was amplified using the universal primers of ITS3 (5′-GCATCGATGAAGAACGCAGC- 3′) and ITS4 (5′ -TCCTCCGCTTATTGATATGC-3′) (20). The Polymerase Chain Reaction (PCR) conditions were as follows: initial denaturation at 95°C for 3 min, 37 cycles of denaturation at 95°C for 30 s, annealing at 53°C for 20 s, elongation at 72°C for 45 s, and final extension at 72°C for 10 min. The integrity and concentration of PCR products were verified by agarose (2%, W/V) gel electrophoresis.

### High-Throughput Sequencing and Bioinformatics Analysis

Purified ITS2 amplicons were sequenced with an Illumina Miseq PE300 platform (Illumina, San Diego, CA, United States). Raw sequences were uploaded to the National Center for Biotechnology Information Sequence Read Archive database with accession numbers SAMN18864574-SAMN18864594.

The quality of demultiplexed reads was checked using Fastp software (v. 0.19.6).^1^ The reads were truncated with a minimum overlap of 10 bp and a PHRED score of at least 20 over a 50-bp sliding window. The reads were clustered into operational taxonomic units (OTUs, 97% similarity) using UPARSE (version 7.0.1090, http://www.drive5.com/uparse/) against the UNITE database (v. 8.0)^2^ in Quantitative Insights Into Microbial Ecology (QIIME, V. 1.9.1)^3^ (21, 22). Then, chimeric sequences were detected and removed by USEARCH (V. 7.0, 7.0).^4^ To ensure the accuracy of OTU annotation, we verified the taxonomical classification of all the OTUs by manual BLAST search in the International Nucleotide Sequence Database Collaboration. We constructed a rarefaction curve for normalization to even depths across samples in QIIME. All the OTUs were denominated at the kingdom, phylum, class, order, family, genus, and species levels. Alpha diversity indices involving Chao1, Good’s coverage, Simpson, ACE, and Shannon were measured through MOTHUR (v. 1.30.2).^5^ In order to estimate beta diversity, weighted UniFrac distance was visualized by principal coordinates analysis (PCoA) and non-metric multidimensional scaling (NMDS). Furthermore, the CF samples were hierarchically clustered by UPGMA. Partial least squares discriminant analysis (PLS-DA) was performed to study the differences between groups using the mixOmics package in the R software. We utilized the linear discriminant analysis effect size (LEFSe) algorithm to analyze the differences in fungal composition between various groups. A Circos graph was constructed using Circos software (23). Venn analysis, community barplot analysis, and heat mapping were conducted using the R software (24). Co-occurrence analysis was applied to reveal interactions among fungal communities at the genus level between different groups with the NetworkX package in Python (25).

## Results

### Diversity of Fungal Community in Crataegi Fructus Samples

**FIGURE 2:**
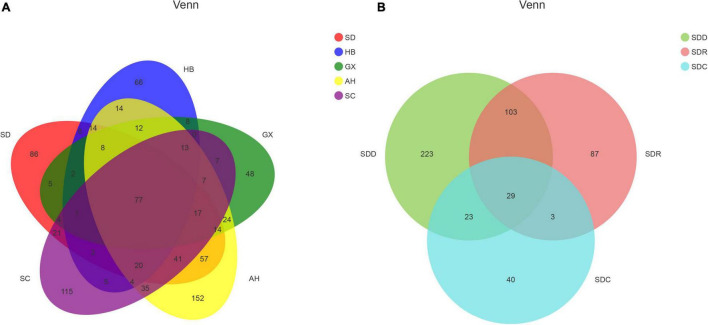
Venn diagram of operational taxonomic units (OTUs) in **(A)** samples from different collection areas and **(B)** samples based on processing methods.

A total of 1,525,316 ITS2 sequences were obtained from 21 CF samples after quality filtering. Rarefaction analysis revealed that the data to determine the depth for each sample was sufficient to estimate the fungal microbiome (Supplementary Figure 1). A total of 925 OTUs were obtained from the 21 CF samples. The distribution of OTUs in the 21 CF samples is listed in Supplementary Table 1. Based on Venn analysis (Figure 2), there were 421 shared OTUs between groups based on various collection areas. The numbers of OTUs in groups based on various collection areas are as follows: AH group 509 OTUs, SD group 378 OTUs, SC group 370 OTUs, HB group 255 OTUs, and GX group247 OTUs. Among the groups based on processing methods, 158 OTUs were shared between different products. The number of OTUs in the SDD group (378 OTUs) was the highest, followed by the SDR (222 OTUs) and SDC (95 OTUs) groups. Five alpha diversity indices, Chao1, Good’s coverage, Simpson, ACE, and Shannon, were measured to estimate the richness and diversity of the fungal community (Supplementary Table 2). High indices of Chao1 and ACE represent a large variation among species. Furthermore, the low Simpson and high Shannon indices demonstrate the high diversity of the fungal community in the samples. The result of Good’s coverage in all samples collected from various areas yielded an estimate of over 99.9%, indicating good overall sampling. Compared with those in the other samples, the Chao1 and ACE indices in the CF11 sample were highest, demonstrating the largest variation in it. In contrast, the highest index of Shannon was observed in the CF15 sample. Among the groups based on processing methods, the index of Good’s coverage in nine samples also yielded an estimate of over 99.9%. The Chao1, Shannon, and ACE indices in the CF3-1 sample were the highest, whereas the Chao1 and ACE indices in the CF1-3 sample were the lowest. Hence, the highest and lowest fungal diversity was observed in the CF3-1 and CF1-3 samples, respectively.

### Composition of Fungal Community in Crataegi Fructus Samples

**FIGURE 3:**
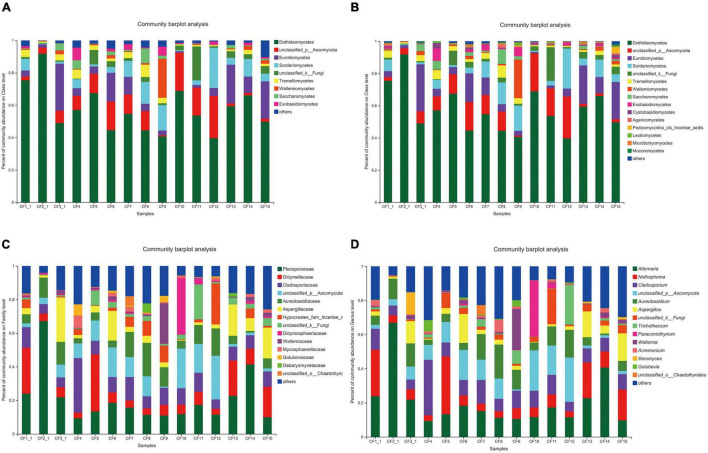
Fungal composition in CF samples from different collection areas at the **(A)** phylum level, **(B)** class level, **(C)** family level, and **(D)** genus level.

All the 925 OTUs taxonomically spanned at least three phyla; some of the OTUs were classified as unclassified fungi or others. Ascomycota was dominant at the phylum level in all the samples that were collected from different areas, with a relative abundance of 64.25–98.09%. The relative abundance of Basidiomycota was 1.45–34.41% (Figure 3A). At the class level, Dothideomycetes (36.70–91.81%) was the most dominant among all the classes (Figure 3B). Furthermore, taxonomical classification at the family level showed that Pleosporaceae (9.35–67.17%) predominated among other families, followed by Didymellaceae (3.34–33.93%) and Cladosporiaceae (5.58–32.46%, Figure 3C). At the genus level, the three most abundant genera were *Alternaria*, *Nothophoma*, and *Cladosporium*, with relative abundances of 9.35–66.92%, 3.35–33.93%, and 5.57–32.46%, respectively (Figure 3D).

**FIGURE 4:**
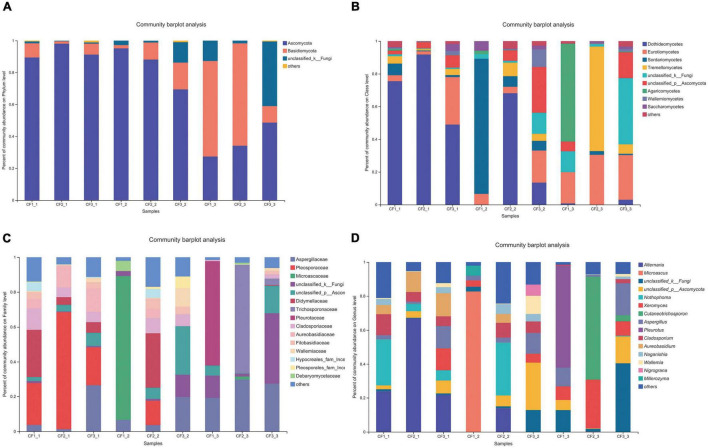
Fungal composition in various processed CF samples at the **(A)** phylum level, **(B)** class level, **(C)** family level, and **(D)** genus level.

As for the groups based on processing methods, Ascomycota (69.44–98.09%) was also dominant at the phylum level in the SDD and SDR groups. However, the relative abundance of Basidiomycota (10.36–64.18%) was higher than that of Ascomycota (27.35–48.61%) in the SDC group (Figure 4A). Dothideomycetes (0–91.81%) was the most abundant at the class level (Figure 4B). Moreover, the abundances of Aspergillaceae, Pleosporaceae, and Microascaceae were highest at the family level, accounting for 1.29–29.74%, 0–67.17%, and 1.67–82.68% (Figure 4C). At the genus level, the relative abundance of *Alternaria* (0–66.92%) was highest among the 207 identifiable genera (Figure 4D).

In addition, among all the 925 OTUs, 135 could be identified at the species level by manual BLAST search. Seven potential mycotoxin-producing fungi were detected, *T. roseum* (detected in CF10, CF2-2, CF11, CF12, CF1-1, CF15, CF2-1, CF3-1, CF14, CF13, CF7, CF5, CF9, CF6, CF4, CF8, CF3-3, CF2-3, CF1-2, and CF1-3), *A. tenuissima* (detected in CF10, CF2-2, CF11, CF12, CF1-1, CF15, CF3-1, CF14, CF13, CF7, CF5, CF6, CF4, and CF8), *A. carbonarius* (detected in CF10, CF2-2, CF11, CF12, CF15, CF2-1, CF3-1, CF13, CF7, CF6, CF8, and CF2-3), *P. brevicompactum* (detected in CF2-2, CF12, CF15, and CF3-1), *A. fumigatus* (detected in CF11, CF15, and CF6), *R. microspores* (detected in CF2-2 and CF3-1), and *P. fermentans* (detected in CF3-1).

### Comparison of Fungal Community in Crataegi Fructus From Different Collection Areas

**FIGURE 5:**
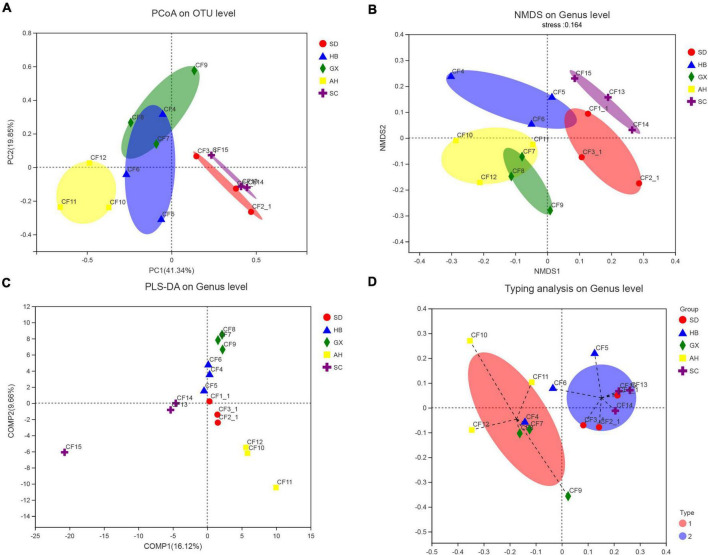
Analysis of the beta diversity of the fungal community in groups based on collection areas. **(A)** Principal coordinates analysis (PCoA) plot on OTU level, **(B)** non-metric multidimensional scaling (NMDS) plot on genus level, **(C)** Partial least squares discriminant analysis (PLS-DA) plot on genus level, and **(D)** typing analysis on genus level.

**FIGURE 6:**
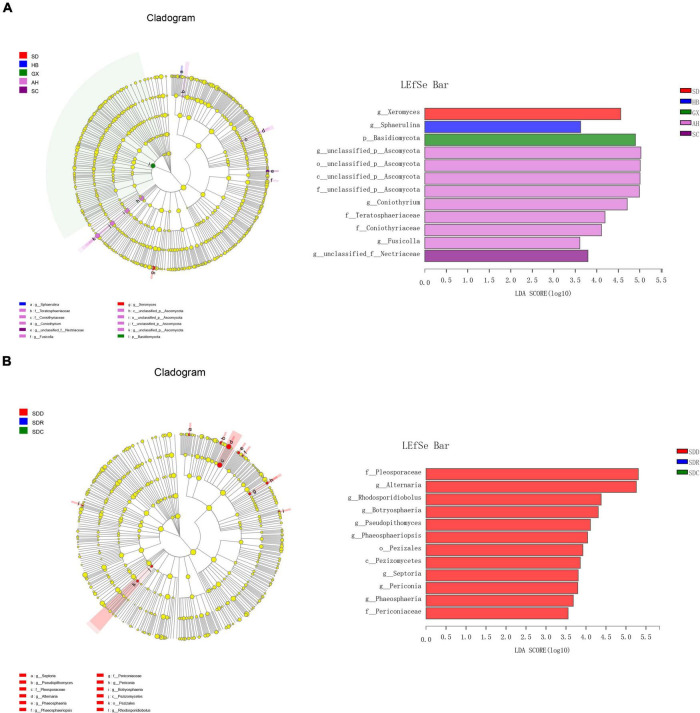
Fungal taxa with different abundances by linear discriminant analysis effect size (LEFSe) analysis in **(A)** groups based on collection areas and **(B)** groups based on processing methods.

**FIGURE 7:**
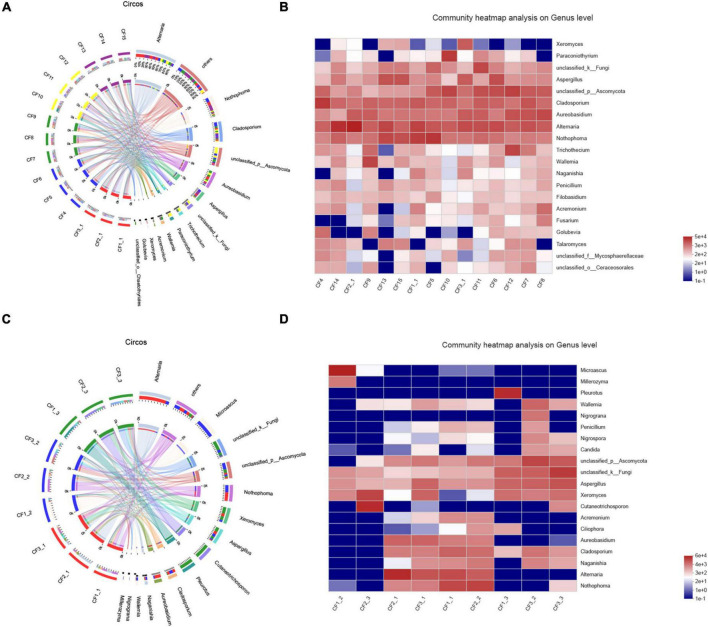
Distribution of fungal community at the genus level in **(A)** samples from different collection areas visualized by Circos, **(B)** a heatmap of the top 20 genera in samples from different collection areas, **(C)** in various processed CF samples, **(D)** a heatmap of the top 20 genera in samples based on processing methods.

We divided the 15 samples into five groups according to collection areas and compared the differences in fungal diversity and composition between different groups. For alpha diversity, the highest Shannon index was detected in the GX group, representing the greatest diversity of the fungal community in this group. Meanwhile, the highest indices of Chao 1 and Ace were observed in the AH group, indicating that this group had the greatest richnes. For beta diversity, we analyzed the diversity of fungal community by PCoA and NMDS analysis. The PCoA result showed that the SC, AH, and SD groups were significantly distinguishable with the exception of the GX and HB groups (ANOSIM, *R* = 0.5467, *P* = 0.001) (Figure 5A). However, differences between the AH and GX groups, and between the SD and HB groups were low based on the NMDS analysis (Figure 5B). Similarly, the PLS-DA result demonstrated that the difference in fungal composition between the SD and HB groups was low (Figure 5C). Furthermore, we compared the differences in fungal communities at various levels between groups (Figure 6A). At the phylum level, the relative abundance of Basidiomycota in the GX group was higher than that in the other groups. At the class level, some unclassified classes belonging to Ascomycota had the highest average percentage of community abundance in the AH group among the five groups. In comparison with the other groups, the average relative abundances of Teratosphaeriaceae, Coniothyriaceae, and some unclassified families belonging to Ascomycota in the AH group were higher. At the genus level, the relative abundance of *Xeromyces* in the SD group was higher than that in the other groups, while the relative abundance of *Sphaerulina* in the HB group was highest among all the groups. Meanwhile, the relative abundances of *Coniothyrium*, *Fusicolla*, and some unclassified genera belonging to Ascomycota were highest in the AH group. In addition, Figures 7A,B show the distribution and differences of the dominant genera with a relative abundance >5% in groups based on collection areas. The relative abundance of *Alternaria*, which was a dominant genus in CF, was higher in the SD and SC groups than in the other groups. We conducted a typing analysis at the genus level (Figure 5D), and the result showed that the relative abundance of *Alternaria* was the main factor that divided the 15 samples into two types, namely, type 1 (AH group, GX group, and CF4) and type 2 (SD group, SC group, CF5, and CF6).

### Comparison of Fungal Community in Groups Based on Processing Methods

**FIGURE 8:**
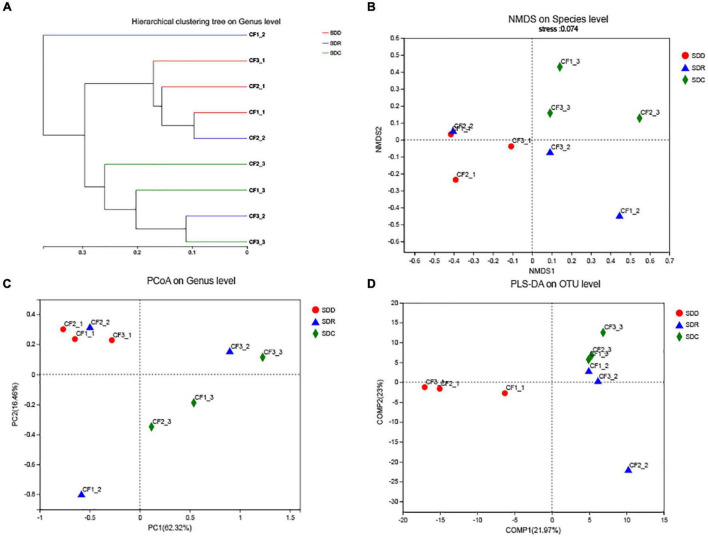
Analysis of the beta diversity of the fungal community in groups based on processing methods. **(A)** Hierarchical clustering tree on genus level, **(B)** NMDS plot on species level, **(C)** PCoA plot on genus level, and **(D)** PLS-DA plot on OTU level.

We collected nine samples and divided them into three groups (SDD, SDR, and SDC) based on processing methods. For alpha diversity, the average indices of Chao 1, Shannon, and ACE were observed to be highest in the SDD group, indicating its high richness and diversity. We also observed that the three groups showed various degrees of clustering based on the result of hierarchical clustering analysis (Figure 8A). Except for the SDR group, the two other groups were clustered into two branches. The results of NMDS analysis and PCoA were also consistent with hierarchical clustering analysis and showed that the SDR samples were assigned to other groups (Figures 8B,C). Based on the PLS-DA result, only the SDC group could be distinguished from the SDD and SDR groups (Figure 8D). With the exception of the SDC group, Ascomycota was the most abundant phylum in the SDD and SDR groups. However, the relative abundance of Basidiomycota was higher than that of Ascomycota in the SDC group. At the class level, the relative abundance of Dothideomycetes was highest in the SDD group, while Eurotiomycetes was the most abundant class in the SDC group. The relative abundance of Pezizales was higher in the SDD group than in the SDR and SDC groups. Based on LEfSe analysis (Figure 6B), the SDD group exhibited higher numbers of Pezizales at the order level and of Pleosporaceae and Periconiaceae at the family level than those in the SDR and SDC group. Meanwhile, *Alternaria*, *Pseudopithomyces*, *Phaeosphaeriopsis*, *Septoria*, *Botryosphareia*, and *Phaeosphaeria* were more common in the SDD group than in the SDR and SDC groups. The fungal composition in various groups showed differences at the genus level. *Alternaria* was dominant in the SDD group, while the relative abundance of *Alternaria* was low in the SDC group. On the contrary, the relative abundance of *Xeromyces* was higher in the SDC group than in the SDD and SDR groups (Figures 7C,D).

### Co-occurrence Analysis

**FIGURE 9:**
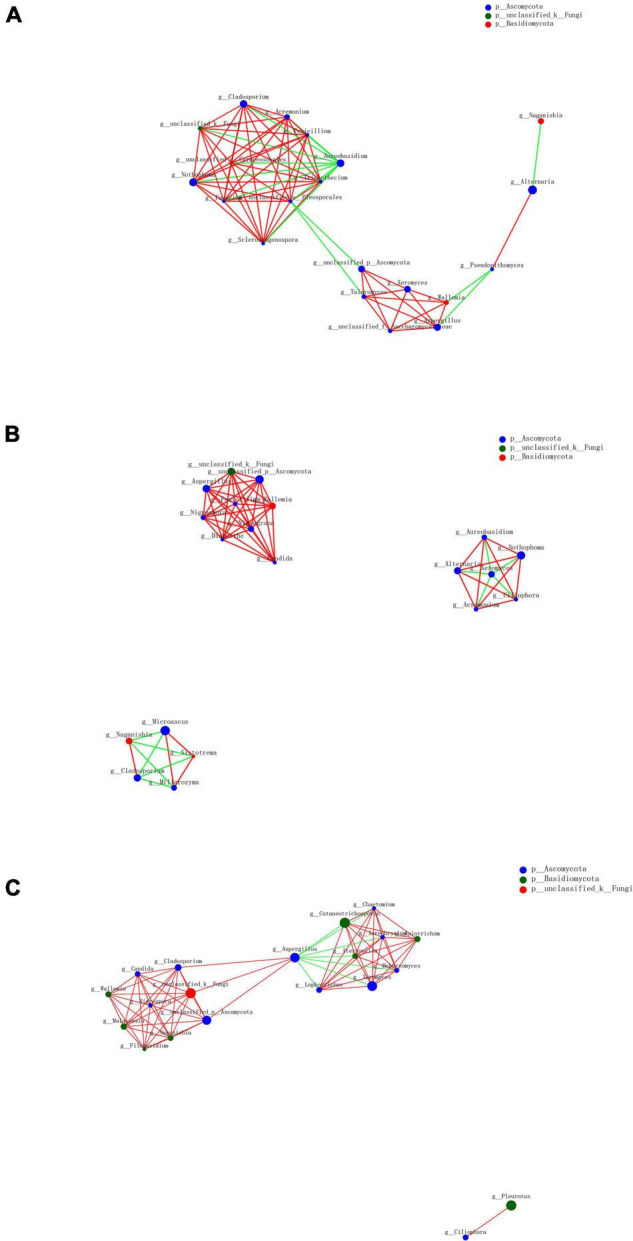
Network analysis of fungal taxa in **(A)** SDD group, **(B)** SDR group, and **(C)** SDC group.

As a result of the diversity of fungal microbiome in different groups based on processing methods, we further analyzed the interactions of fungal genera among various groups. The top 20 genera were selected to reveal the microbiome relationship. The co-occurrence network analysis showed that the difference was distinguishable depending on different processing methods (Figure 9). A total of 55 positive and 14 negative correlations were recorded in the SDD group, while 51 positive and 11 negative correlations were recorded in the SDR group. Meanwhile, the SDC group had 63 positive and 7 negative correlations. The result showed that *Alternaria*, which was the dominant genus, was positively correlated with *Pseudopithomyces* but negatively correlated with *Naganishia* in the SDD group, while *Alternaria* was negatively correlated with *Xeromyces* and positively correlated with *Aureobasidium*, *Nothophoma*, *Ciliophora*, and *Acremonium* in the SDR group. Except for *Alternaria*, the correlations of *Xeromyces* differed among the three groups. In the SDD group, *Xeromyces* showed positive correlations with *Wallemia*, *Aspergillus*, *Talaromyces*, and some unclassified genera belonging to the Saccharomycetaceae family. However, *Xeromyces* exhibited negative correlations with *Aureobasidium*, *Nothophoma*, *Ciliophora*, *Acremonium*, and *Alternaria*. Furthermore, this genus was negatively correlated with *Aspergillus* and positively correlated with *Lophotrichus*, *Itersonilia*, *Cutaneotrichosporon*, *Chaetomium*, *Xerochrysium*, *Apiotrichum*, and *Dearyomyces*. In addition, *Aspergillus* exhibited the most correlations (10 correlations) in the SDC group, followed by the SDR (eight correlations) and SDD (six correlations) groups. For *Wallemia*, more correlations (eight correlations) were detected in the SDR group than in the SDC (seven correlations) and SDD (five correlations) groups. However, *Cladosporium* had a higher number of correlations in the SDD and SDC groups (nine correlations) than in the SDR group (four correlations).

## Discussion

### Fungal Contamination in Crataegi Fructus

Crataegi Fructus is considered a popular dietary supplement that has attracted global attention as a result of its edible and medicinal effects. However, exogenous contaminations, especially fungal contamination, in CF are inevitable. In this study, we detected fungal contamination in 21 CF samples that were collected in China. The result showed that all the samples were contaminated with fungi. At the phylum level, Ascomycota was dominant among most of the samples (except for CF1-3 and CF2-3). It is a common phylum that has high metabolite diversity (26). Meanwhile, this phylum also includes a wide range of plant and animal pathogens such as species from *Alternaria*, *Aspergillus*, *Penicillium*, and *Fusarium*. Contamination of foods and herbs by Ascomycota has been reported continuously. Zang et al. studied the dynamics of microbial community during fermentation of Suan yu (fermented fish) and found that Ascomycota was the predominant phylum in all stages of fermentation (27). In Saudi Arabia, Hashem and Alamri isolated 520 fungal strains from 15 spices, indicating that most of the species belonged to Ascomycota (28). In addition, it was observed that *Alternaria* contamination was detected in 16 out of 21 CF samples in this study, showing that *Alternaria* was the main fungal genus in the CF samples. According to a relevant study, *Alternaria* is a common and important fungal genus that is widely distributed worldwide. It is divided into 24 sections based on morphological and molecular identification (29). Many species in this genus have been considered as plant and post-harvest pathogens such as *Alternaria bataticola*, *Alterneria porri*, and *Alterneria solani* (30, 31). A previous study has reported that contamination by *Alternaria* species caused damage in *Crataegus* sp. tree leaves (32), while few studies focused on the contamination by *Alternaria* in CF fruits. Our result indicated that *Alternaria* was the most abundant genus among all the genera that contaminated CF. Similarly, *Alternaria* has also been reported as the main contaminated fungal genus in other herbs. The study performed by Pickova et al. showed that the quality of Milk Thistle, which is an herb used for treatment of liver disease, was affected by fungi in *Alternaria* and their mycotoxins (33). Zhao et al. also indicated that fungal strains in *Alternaria* severely threatened the industry of *Dendrobium officinale* (34). Furthermore, several *Alternaria* species were not only considered as spoilage agents for herbs but were also capable of synthesizing some mycotoxins such as alternariol, alternariol monomethyl ether, and altenuene, causing damage to human health (35). Thus, in order to identify the fungi in *Alternaria* efficiently and accurately, an identification method deserves to be developed. HTS provides an efficient method for the efficient identification of fungi that contaminate CF, guaranteeing safe utilization of CF.

### Effect of Processing Method on the Fungal Community in Herb and Food

In order to improve efficacy and decrease toxicity, many herbs are processed before being distributed to the market. However, the processing methods also affect the fungal contamination in herbs. According to the Chinese Pharmacopoeia (2020), CF can be processed into dried, roasted, and charred products, and their efficacy shows degrees of differences in clinics. Moreover, few studies reported a comparison of the fungal microbiome in these products. In this study, we compared the difference in fungal composition and diversity between processed CF products. The result showed differences in the fungal community in the three groups. At the phylum level, Ascomycota was dominant in the SDD and SDR groups, while the relative abundance of Basidiomycota was highest in the SDC group. Furthermore, remarkable differences were observed at the genus level. According to the fungal composition analysis, the relative abundances of *Aspergillus* and *Xeromyces* were highest in the SDC group, followed by the SDR and SDD groups, and the relative abundances of *Alternaria* and *Aureobasidium* were highest in the SDD group, followed by the SDR and SDC groups. It can be concluded that the fungal community in the CF samples changes as the effect of processing temperature increases. Some fungal genera such as *Aspergillus* and *Xeromyces* become dominant from low abundance, while others such as *Alternaria* and *Aureobasidium* decrease significantly or are not even detected in the samples. During the CF processing procedure, temperature is an important factor that may affect the fungal community. Based on previous studies, temperature has a significant impact on fungal growth and mycotoxin production. Mellon et al. assessed the effect of temperature on the growth and aflatoxin production of *A. flavus* and *A. parasiticus*. The results showed that high temperature exhibited more inhibitory effect on the growth and aflatoxin production of these fungi than low temperature (36). Similarly, Cibelli et al. observed that the growth of *A. alternata* was remarkably inhibited under high-temperature conditions (37). Moreover, many studies have reported the impact of other processing methods on the fungal community. Cao et al. analyzed the variation of microbial community during the fermentation of Huafeng Dan Yaomu and found that the dominant fungal genera changed from *Millerozyma* and *Saccharomycopsis* to *Pichia* after 14 days of fermentation (38). In Nigeria, Omohimi et al. collected yam samples (including raw samples, chips, flakes, and flour) to compare the difference in fungal contamination. The result showed that the frequency of some strains in *Aspergillus* and *Fusarium* in processed samples was higher than that in dried samples (39). In addition to herbs, many foods will be processed to improve their taste or extend shelf life, such as hams. Mu et al. observed that the fungal community in Panxian Ham changed during the processing procedure (selection, salting, resting, and drying-ripening) (40), and this result was similar to the studies performed by Zhang et al. (41). In conclusion, the processing procedure influences the fungal community in herbs and foods significantly. Thus, it is important to study the relationship between processing methods and the fungal community, providing references for industrial processors to construct standard quality parameters.

### Molecular Identification of Toxigenic and Non-toxigenic Strains

In this study, seven potential mycotoxin-producing fungi were detected in the CF samples, including *T. roseum*, *A. tenuissima*, *A. carbonarius*, *P. brevicompactum*, *A. fumigatus*, *R. microspores*, and *P. fermentans*. Previous studies reported that all these fungi were capable of producing mycotoxins, such as zearalenone, T-2 toxin, and deoxynivalenol (42–47). However, these fungi exhibit mycotoxin-producing ability inconsistently and are often non-toxigenic under some conditions. Both external and internal factors may influence the synthesis of mycotoxin (48–50). Therefore, the molecular identification of toxigenic and non-toxigenic fungi has attracted the attention of researchers. Kim et al. applied multiplex PCR to detect aflatoxigenic and non-aflatoxigenic fungi by designing two primer sets, primer set I (omtB/alfR/omtA) and primer set II (alfR/ver-1/omtA), indicating that only aflatoxigenic *Aspergillus* species could show three patterns in both primer sets (51). Similarly, Davari et al. characterized the aflatoxigenic *A. flavus* and *Aspergillus parasiticus* strains successfully using four sets of primers (specifically for nor1, ver1, omtA, and aflR) (52). Norlia et al. observed that all aflatoxin biosynthesis genes (*aflR*, *aflP*, *aflD*, *aflM*, and *pksA*) were amplified in aflatoxigenic *A. flavus*, and that at least one of these genes could not be amplified in non-aflatoxigenic *A. flavus* (53). Thus, a molecular method for efficient and accurate detection of toxigenic fungi should be developed to provide early warning and ensure the microbiological safety of herbs.

## Conclusion

In this study, fungal contamination in CF was investigated using ITS2 amplicon sequencing. All the samples were detected to be positive for fungal contamination. A total of 115 fungal species were identified, among which seven were potential mycotoxin-producing fungi. Moreover, HTS has been demonstrated as a feasible method for efficient detection of fungal contamination in CF, serving as a consolidate reference for its safe application and quality improvement.

## Data Availability Statement

The datasets presented in this study can be found in online repositories. The names of the repository/repositories and accession number(s) can be found below: https://www.ncbi.nlm.nih.gov/genbank/, SAMN18864574-SAMN18864594.

## Author Contributions

XP: conceptualization, funding acquisition, resources, and validation. JY and MG: data curation. JY, MG, WJ, YD, and XP: formal analysis. JY and XP: methodology, writing (original draft), and writing (review and editing). All authors contributed to the article and approved the submitted version.

## Conflict of Interest

The authors declare that the research was conducted in the absence of any commercial or financial relationships that could be construed as a potential conflict of interest.

## Publisher’s Note

All claims expressed in this article are solely those of the authors and do not necessarily represent those of their affiliated organizations, or those of the publisher, the editors and the reviewers. Any product that may be evaluated in this article, or claim that may be made by its manufacturer, is not guaranteed or endorsed by the publisher.

## Footnotes

1
https://github.com/OpenGene/fastp

2
https://unite.ut.ee/

3
http://qiime.org/install/index.html

4
http://www.drive5.com/usearch/

5
https://www.mothur.org/wiki/Download_mothur

## Supplementary Material

Supplementary Figure 1 Rarefaction curves for operational taxonomic unit (OTU) in each Crataegi Fructus (CF) sample.

Supplementary Table 1 Distribution of operational taxonomic units (OTUs) for 21 Crataegi Fructus (CF) samples.

Supplementary Table 2 Alpha diversity indices in each CF sample.
