# Supplementary figures and images for: Illumina-Based Analysis Yields New Insights Into the Fungal Contamination Associated With the Processed Products of Crataegi Fructus
Source: Front Nutr. 2022 May 12;9:883698. doi: 10.3389/fnut.2022.883698 (PMC9135361; doi:10.3389/fnut.2022.883698)

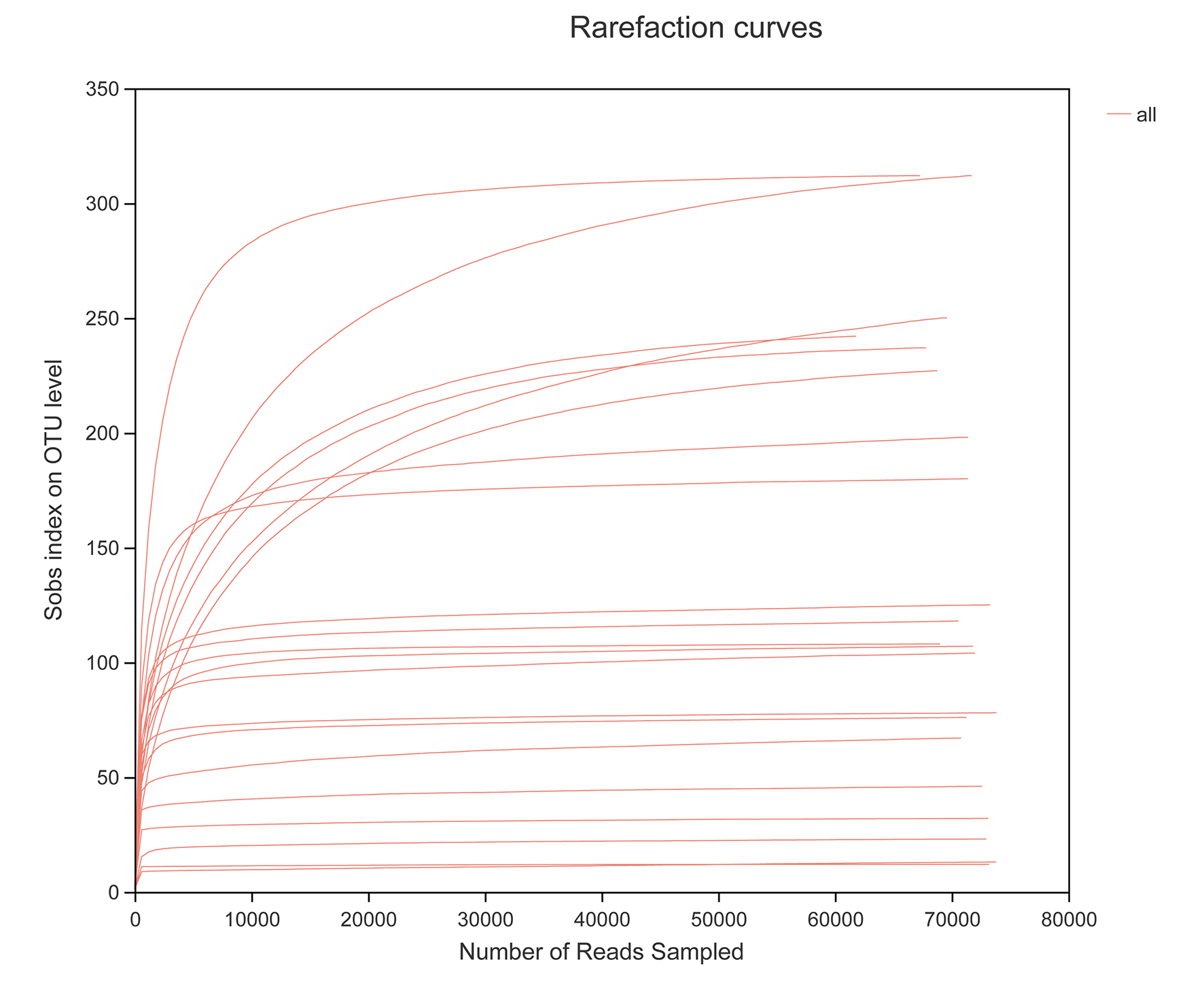

Supplement: Supplementary Figure 1 — Rarefaction curves for operational taxonomic unit (OTU) in each Crataegi Fructus (CF) sample. [file Image_1.TIF]
